# Correlation between the atherogenic index of plasma and risk of diabetic retinopathy

**DOI:** 10.3389/fmed.2025.1565414

**Published:** 2025-05-16

**Authors:** Yuan Zhang, Guanhua Chen, Yali Jing

**Affiliations:** ^1^Department of Endocrinology, Endocrine and Metabolic Disease Medical Center, Nanjing Drum Tower Hospital Clinical College of Nanjing University of Chinese Medicine, Nanjing, China; ^2^Branch of National Clinical Research Centre for Metabolic Diseases, Nanjing, China

**Keywords:** type 2 diabetes mellitus, atherogenic index of plasma, diabetic retinopathy, predictor, biomarker

## Abstract

**Objective:**

To investigate the relationship between the atherogenic index of plasma (AIP) and diabetic retinopathy (DR) in patients with type 2 diabetes mellitus (T2DM).

**Methods:**

A total of 584 patients with T2DM were divided into two groups based on whether with DR (non-DR group, *n* = 382; DR group, *n* = 202). The association between AIP and DR was assessed by Spearman’s correlation and bivariate/multivariate logistic regression.

**Results:**

The patients in the DR group showed significantly higher AIP levels than those in the non-DR group (−0.009 ± 0.226 vs. 0.186 ± 0.261, *p* < 0.001). Compared with those without DR, DR group had higher levels of age, systolic blood pressure (SBP), body mass index (BMI), diabetes duration, triglycerides (TG), blood urea nitrogen (BUN) and creatinine (Cr), while direct bilirubin (DBIL) and high-density lipoprotein cholesterol (HDL-C) were lower (*p* < 0.05). According to the interquartile range of AIP, the participants were divided into four groups: Q1 (≤−0.130), Q2 (−0.129, 0.048), Q3 (0.049, 0.220), Q4 (≥0.221). After adjusting for age, BMI, SBP, diabetes duration, DBIL, BUN and Cr, the logistic regression model indicated that subjects in Q3 and Q4 still had a remarkably increased risk of DR (Q3: OR, 2.838, 95% CI: 1.268 ~ 7.067; Q4: OR, 4.414, 95% CI: 1.841 ~ 10.097; all *p* < 0.05). AIP provided an AUC value of 0.697 for retinopathy in patients with T2DM (95% CI: 0.652 ~ 0.741).

**Conclusion:**

AIP is associated with diabetic retinopathy in patients with T2DM, and AIP may be a potential predictor of DR in patients with T2DM.

## 1 Introduction

With the intensification of global aging and changes in lifestyle, the incidence of diabetes mellitus (DM) is on an increasing trend, 90% of which is type 2 diabetes mellitus (T2DM) (1). Diabetic retinopathy (DR) is one of the common microvascular complications of diabetes mellitus and is the main cause of vision impairment and loss in diabetic patients. It is estimated that the prevalence of DR is 22.27% in DM; and by 2045, the number of people with DR will grow from 103.12 million in 2020 to 160.5 million (2). DR is associated with a higher risk of peripheral neuropathy, cardiovascular events, and fracture events, severely affecting patients’ physical and mental health and quality of life.

Dyslipidemia, characterized by disorders of lipid metabolism involving abnormally elevated levels of total cholesterol (TC), triglycerides (TG), low-density lipoprotein cholesterol (LDL-C), and lowered levels of high-density lipoprotein cholesterol (HDL-C), has been widely studied and is strongly associated with the onset and progression of T2DM (3). Significantly, previous studies also have shown that patients with diabetes combined with dyslipidemia have a higher risk of developing DR, and a higher incidence of non-proliferative diabetic retinopathy (NPDR), diabetic macular edema (DME) and proliferative DR. In addition, statin therapies used to control dyslipidemia have been associated with a lower incidence of DR and a reduced need for treatment of sight-threatening DR. (4–8) Considering the importance of lipid metabolism in the pathogenesis of DR, it is beneficial to pay attention to changes in lipid-related factors in T2DM patients for clinical prediction of DR.

In clinical practice, it is not sufficient to diagnose risk stratification for DR based on a single lipid marker due to differences in factors such as the duration of the patient’s disease and the number of co-morbidities. Therefore, we hypothesized that using a combination of lipid indices might provide a more comprehensive assessment of lipid status than relying on a single lipid factor. Atherogenic index of plasma (AIP) as a novel composite lipid index, defining as the logarithm of the ratio of TG to HDL-C, represents a great marker in the cardiovascular diseases and was observed to be correlated closely with LDL-C particle size (9, 10). AIP improves sensitivity to dynamic metabolic abnormalities by integrating two lipid parameters—TG and HDL-C—a nonlinear transformation. It was an independent risk factor for CVD in diabetic patients and was validated by multiple models (11–13). The incidence of cardiovascular disease (CVD) in T2DM patients is 2–3 times higher than in non-T2DM, partly attributed to the dyslipidemia accompanies T2DM (14). Moreover, a meta-analysis showed that AIP was a more accurate predictor of diabetes risk compared to other lipid components (15). Therefore, in the present study, we sought to explore the relationship between the composite lipid index AIP and DR occurrence in a cross-sectional study. We aimed to corroborate whether AIP is an independent predictor of DR occurrence. The findings of this study are anticipated to bridge the gap between DR and markers of composite lipid function and have clinical implications for the management of DR.

## 2 Materials and methods

### 2.1 Study design and population

This study was a cross-sectional study. We retrospectively collected patients with T2DM, aged between 18 and 70 years, who were hospitalized in the Endocrinology Department of Nanjing Drum Tower Hospital between January 2016 and January 2018. T2DM was diagnosed according to the 2003 American Diabetes Association criteria (16). The International Clinical Diabetic Retinopathy Scale was used to group the presence or absence of retinopathy (17). Based on the presence or absence of DR, the study subjects were categorized into DR group (DR group) and non-DR group (non-DR group). Subjects with combined acute complications of diabetes mellitus, severe infections, dysfunction of vital internal organs and tissues (heart, liver, kidney, etc.), and malignant tumors were excluded. Patients with hormonal or additive medications within the last 3 months that could have an effect on endocrine metabolism were also excluded.

### 2.2 Data collection

General clinical information such as age, height, weight and blood pressure levels were recorded. And calculate body mass index (BMI) = weight (kg)/ height (m^2^). Biochemical measurements were performed after fasting for at least 10 h. The enzymatic auto-analyzer (Kyowa Medex Co., Ltd., Tokyo, Japan) was used to measure concentrations of fasting plasma glucose (FPG), triglyceride (TG), total cholesterol (TC), high-density lipoprotein cholesterol (HDL-C), and low-density lipoprotein cholesterol (LDL-C), following the manufacturer’s instructions. Calculate atherogenic index of plasma (AIP) = log (TG/HDL-C) (10).

### 2.3 Statistical analysis

After normality testing, the mean ± standard deviation (SD) was utilized to describe the continuous parameters in a normal distribution, otherwise shown as median [interquartile range (IQR)]. For variables in a normal distribution, t-test was employed to compare the difference between two groups. Otherwise, the Mann–Whitney U test was used for analysis. Categorical parameters were described as percentages and numbers. The chi-square (*χ*^2^) test or Fisher’s exact test was used for comparisons of categorical variables. Spearman correlation analysis was used to explore the correlation between AIP and each variable. Binary logistic regression was used to analyze the overall risk of DR by different levels of AIP. The results were evaluated within a 95% confidence interval (CI) and at a significance level of two-sided *p*-value less than 0.05. All data were examined by adopting the SPSS 27.0 software (SPSS Inc., IBM) and R 4.2.2. Statistics were considered significant when *p* < 0.05.

## 3 Results

**Figure 1:**
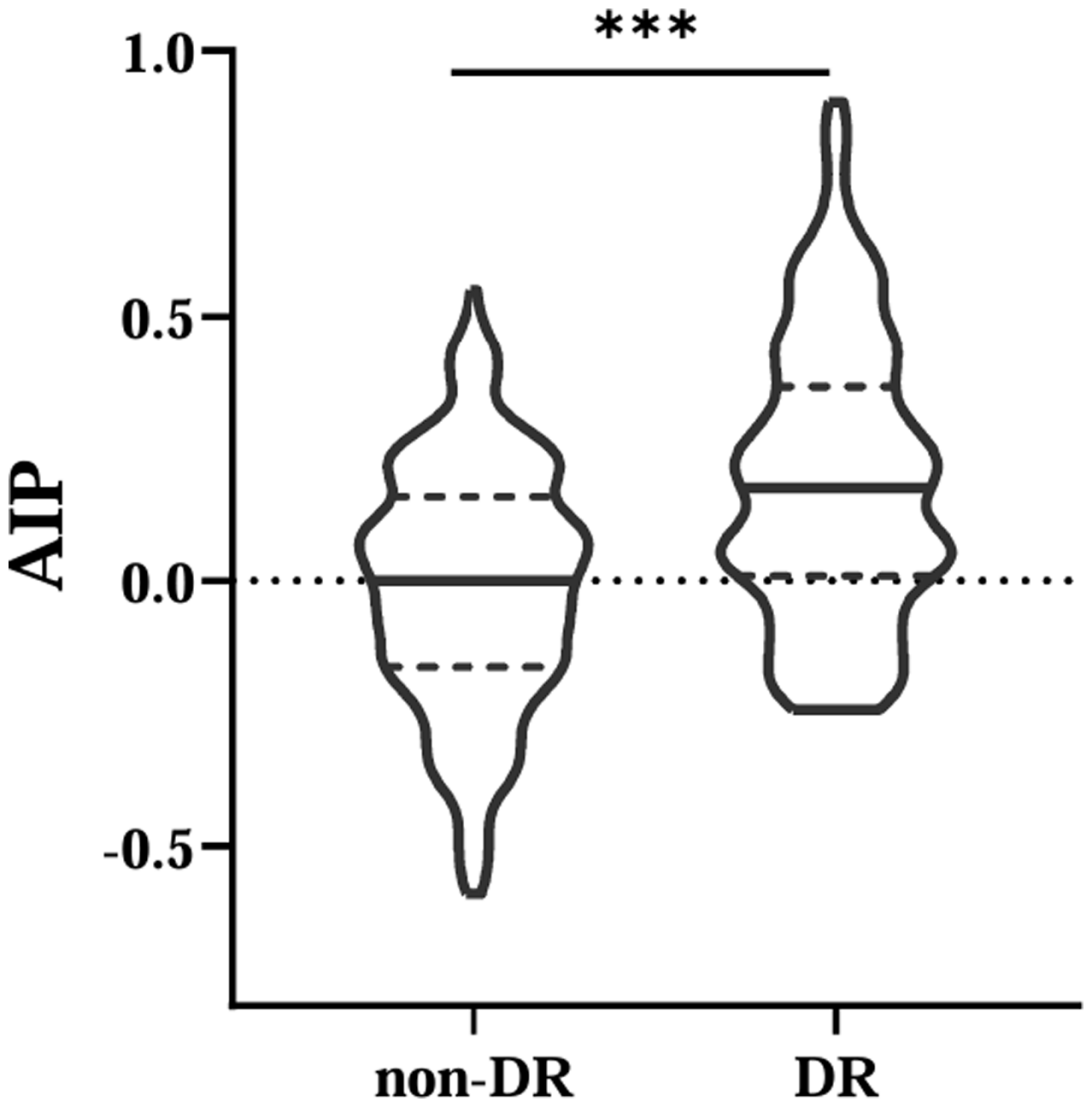
Comparisons of AIP between non-DR and DR groups. AIP, atherogenic index of plasma. ***Denotes significance at a *p* value of <0.001.

This study included 584 patients with T2DM (390 males and 194 females), 202 patients with DR. The results showed that AIP values were higher in the DR group patients (*p* < 0.001) (Figure 1). Moreover, compared with those without DR, participants with DR had higher levels of age, systolic blood pressure (SBP), BMI, duration, TG, blood urea nitrogen (BUN) and creatinine (Cr); while direct bilirubin (DBIL) and HDL-C were lower (all *p* < 0.05). There were no statistically significant differences in other parameters (*p* > 0.05) (Table 1).

**Table 1:** Baseline characteristics of participants between non-DR and DR groups.

Variables	Total (*n* = 584)	Non-DR (*n* = 382)	DR (*n* = 202)	*χ*^2^/F/H	*p*-value
Age (year)	56.65 (52.00, 63.00)	54.08 (48.00, 62.00)	61.49 (56.00, 68.00)	−10.412	<0.001
Male (n, %)	383, 65.6%	261, 66.9%	121, 62.4%	1.186	0.310
SBP (mmHg)	131.87 ± 15.88	130.64 ± 16.06	134.33 ± 15.33	0.096	0.006
DBP (mmHg)	79.13 ± 11.09	79.19 ± 11.00	79.03 ± 11.28	0.048	0.880
BMI (kg/m2)	24.58 ± 2.96	24.26 ± 2.77	25.25 ± 3.18	1.303	<0.001
Duration (year)	9.44 (4.00, 14.00)	8.87 (4.00, 13.00)	10.55 (5.00, 16.00)	−3.069	0.003
FPG (mmol/L)	8.12 ± 2.43	8.05 ± 2.33	8.23 ± 2.60	1.508	0.404
2 h-PG (pmol/L)	14.31 ± 4.38	14.21 ± 4.55	14.56 ± 4.02	2.325	0.386
HbA1c (%)	8.65 (6.90, 10.10)	8.64 (6.90, 10.10)	8.66 (7.00, 10.00)	−0.106	0.937
AST (U/L)	21.15 (15.10, 23.10)	21.10 (14.75, 23.15)	21.47 (16.00, 23.05)	−0.988	0.765
ALT (U/I)	26.22 (15.30, 29.60)	26.67 (14.90, 29.60)	25.90 (15.90, 29.55)	−1.566	0.631
TBIL (umol/L)	13.07 (9.30, 15.10)	12.89 (9.55, 15.55)	13.49 (8.88, 14.08)	−1.723	0.603
DBIL (umol/L)	3.86 (2.80, 4.60)	4.00 (3.00, 4.65)	3.63 (2.50, 4.43)	−2.499	0.048
TG (mmol/l)	1.39 (0.91, 1.68)	1.20 (0.84, 1.47)	1.75 (1.06, 2.28)	−7.589	<0.001
TC (mmol/l)	4.23 ± 1.06	4.21 ± 1.04	4.28 ± 1.08	1.218	0.404
HDL-C (mmol/l)	1.13 ± 0.29	1.17 ± 0.29	1.04 ± 0.25	2.580	<0.001
LDL-C (mmol/l)	2.49 (1.81, 3.10)	2.47 (1.83, 3.07)	2.53 (1.78, 3.14)	−0.922	0.477
AIP	0.059 ± 0.256	−0.009 ± 0.226	0.186 ± 0.261	5.993	<0.001
BUN (mmol/l)	5.66 (4.70, 6.30)	5.46 (4.70, 6.10)	6.03 (4.85, 6.60)	−2.675	0.044
Cr (umol/l)	60.94 ± 14.76	59.74 ± 13.74	63.21 ± 16.28	3.809	0.011
UA (umol/l)	330.13 ± 129.95	325.95 ± 147.62	338.01 ± 86.30	0.308	0.216

BMI, body mass index; SBP, systolic blood pressure; DBP, diastolic blood pressure; FPG, fasting plasma glucose level; 2 h-PG, 2-h postprandial blood glucose; HbA1c, glycated hemoglobin A; ALT, alanine aminotransferase; AST, aspartate aminotransferase; TBIL, total bilirubin; DBIL, direct bilirubin; TG, triglycerides; TC, total cholesterol; HDL-C, high-density lipoprotein cholesterol; LDL-C, low-density lipoprotein cholesterol; BUN, blood urea nitrogen; Cr, creatinine; UA, uric acid.

**Figure 2:**
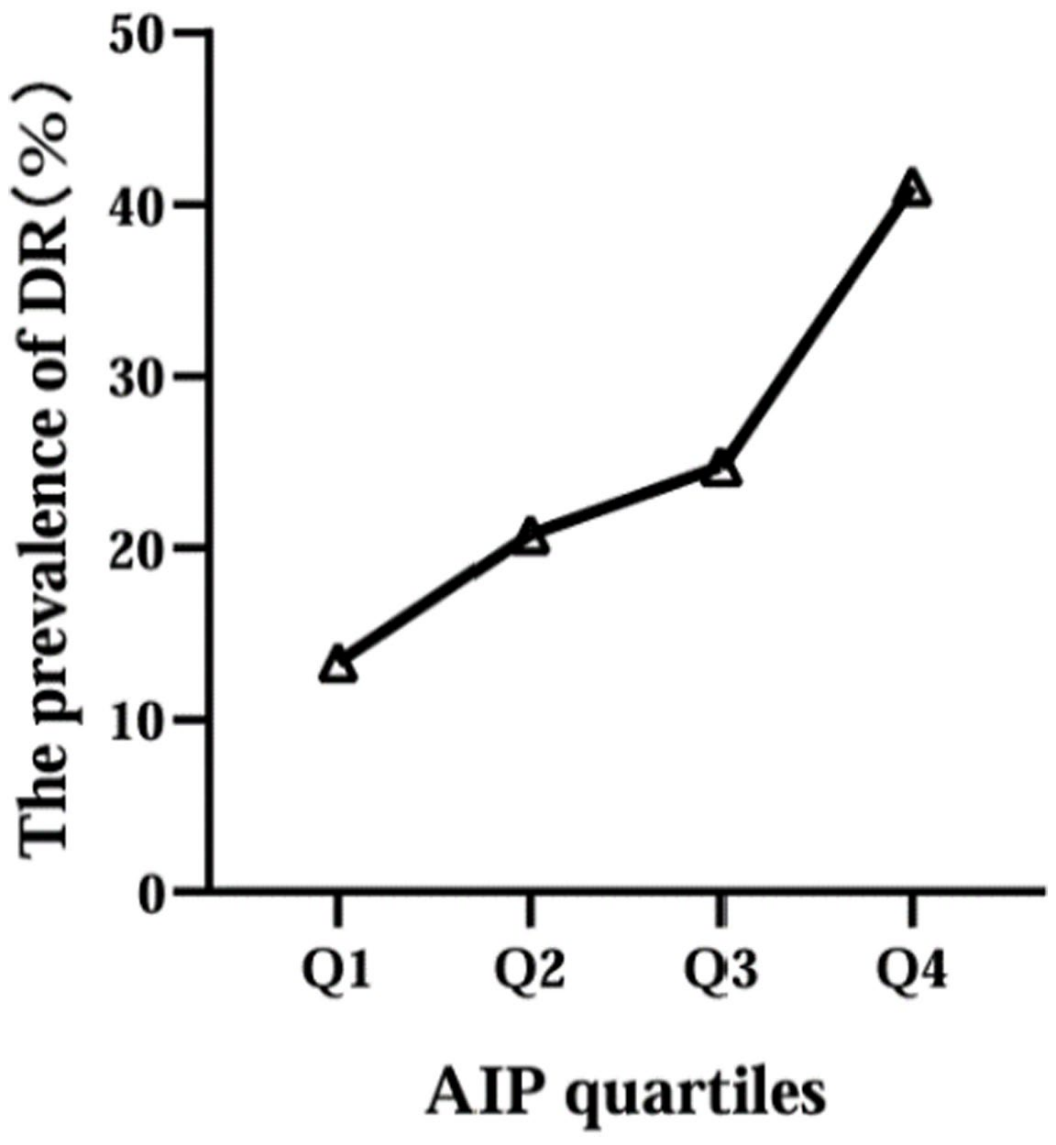
Relationship between AIP quartile and the prevalence of DR.

Then, according to the interquartile range of AIP, the participants were divided into four groups: Q1 (≤−0.130), Q2 (−0.129, 0.048), Q3 (0.049, 0.220), Q4 (≥0.221). We found that the prevalence of DR increased with increasing AIP levels, 27 in Q1 group (19.6%), 42 in Q2 group (28.6%), 50 in Q3 group (34.2%) and 83 in Q4 group (56.8%) (Figure 2).

**Figure 3:**
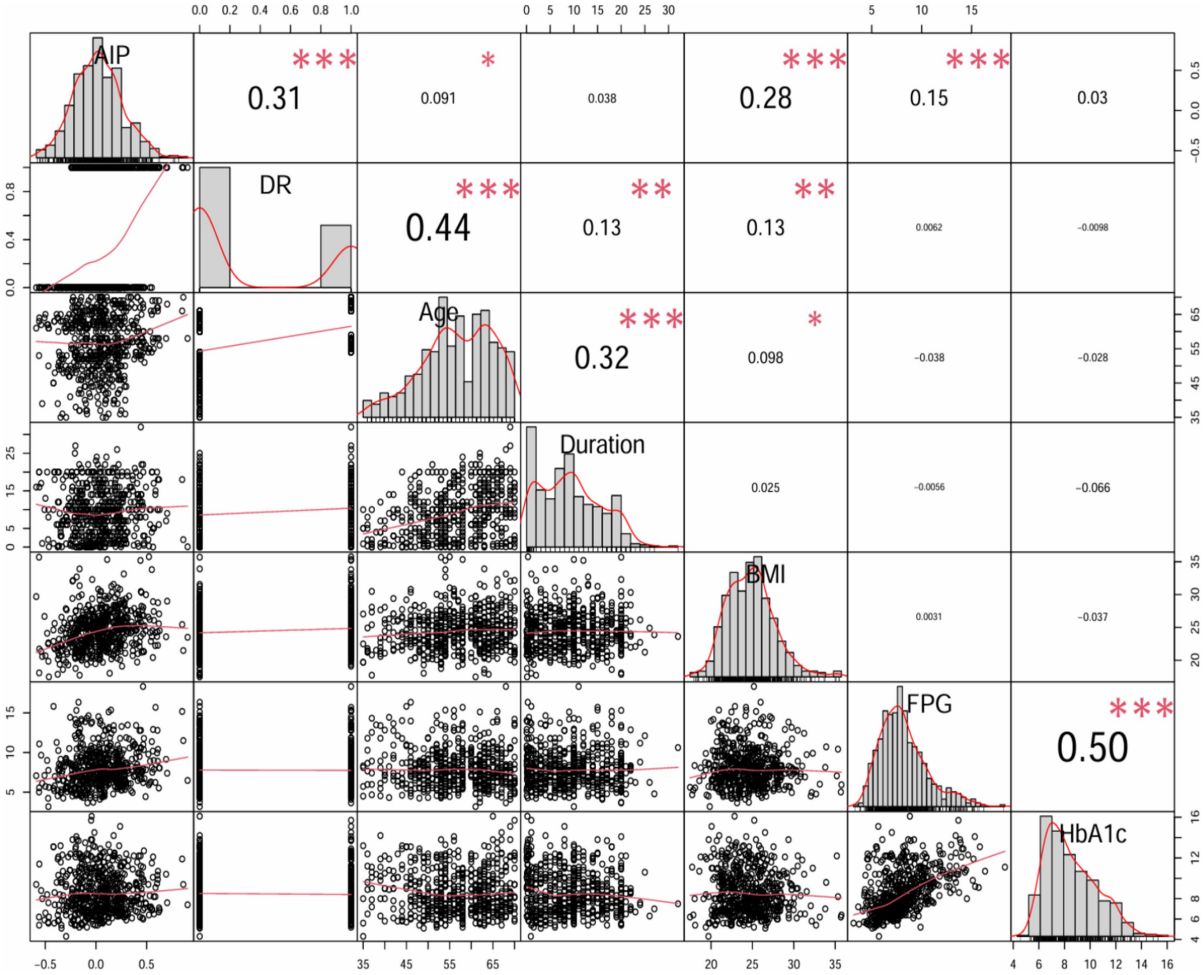
Matrix scatter diagram by Spearman’s test for AIP, DR, age, duration, BMI, FPG and HbA1c.

Moreover, spearman’s rank correlation coefficient analysis was utilized to analyze the effect of AIP on the incidence of DR and some important variables. AIP was significantly positive correlated with DR (r = 0.31, *p* < 0.001), age (r = 0.091, *p* < 0.05), BMI (r = 0.28, *p* < 0.001) and FPG (r = 0.15, *p* < 0.001); however, there was no significant correlation between AIP and diabetes duration or HbA1c (Figure 3).

**Figure 4:**
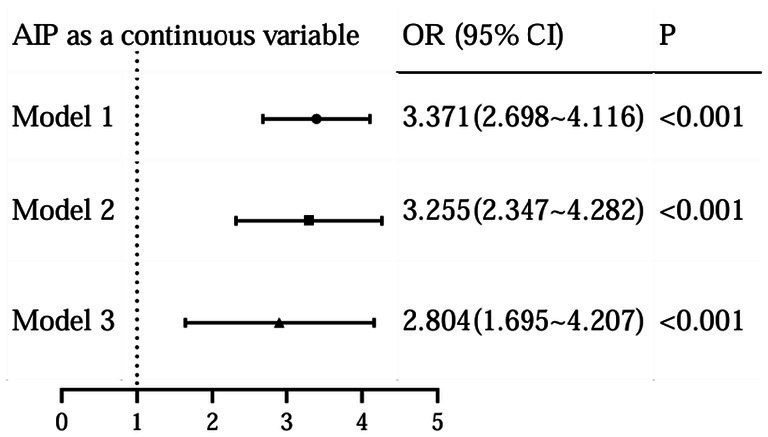
Logistic regression for DR with AIP as a continuous variable.

**Figure 5:**
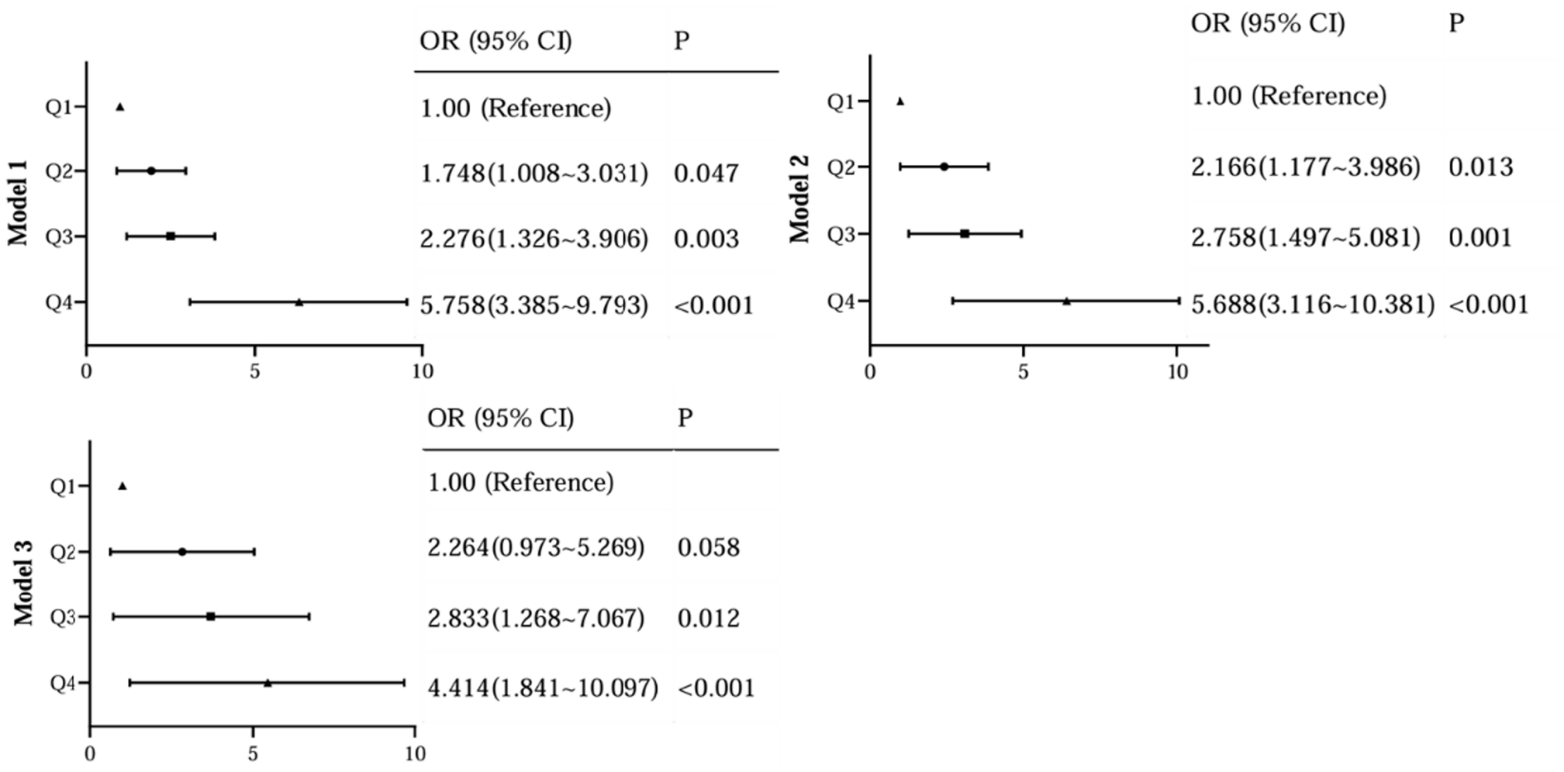
Regression analysis of AIP as a stratification variable with DR.

To further assess the relationship between the levels of AIP and the occurrence of DR, we performed a binary logistic analysis. In regression analysis of AIP as a continuous variable, it was shown that AIP levels were significantly associated with DR both in unadjusted and adjusted models for all subjects in this study (Figure 4). With the AIP as a stratified indicator, in unadjusted model (model 1) compared with Q1, Q2 (Q2 vs. Q1, OR, 1.748; 95% CI: 1.008 ~ 3.031), Q3 (Q3 vs. Q1, OR, 2.276; 95% CI: 1.326 ~ 3.906) and Q4 (Q4 vs. Q1, OR, 5.758; 95% CI: 3.385 ~ 9.793) all kept an independent effect on DR presence (Figure 5). After age and BMI adjustment, in comparison to Q1group, patients in Q2, Q3 and Q4 all had a significantly increased risk of DR by 116.6% (OR, 2.166; 95% CI:1.117 ~ 3.986), 175.8% (OR, 2.758; 95% CI:1.497 ~ 5.081) and 468.8% (OR, 5.688; 95% CI:3.116 ~ 10.381) respectively (Figure 5). After further adjustment for SBP, duration, DBIL, BUN and Cr, compared to the Q1 of AIP, subjects in Q3 and Q4 still had a remarkably increased risk of DR (OR 2.838, 4.414, separately) (Figure 5).

**Figure 6:**
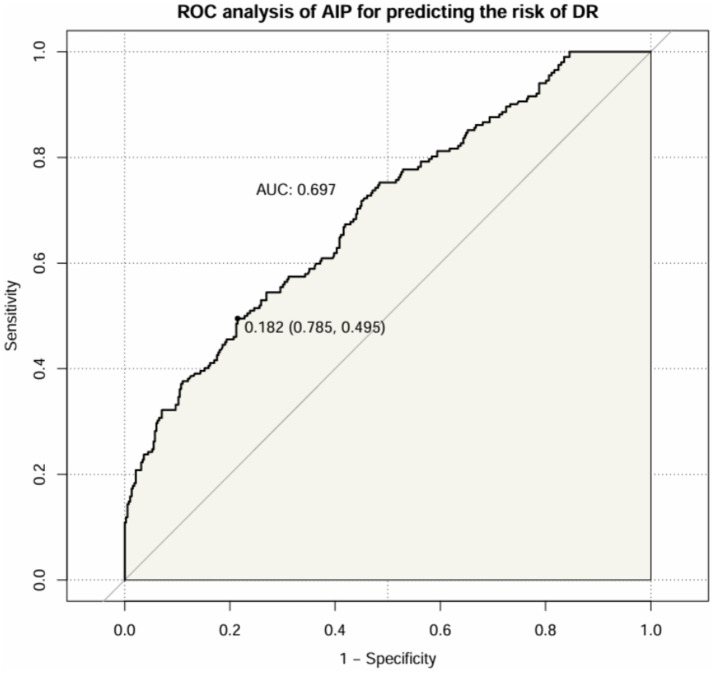
ROC curve of AIP index predicting DR in patients with T2DM.

After that, we assessed the diagnostic value of AIP for DR in patients with T2DM (Figure 6). The results showed that the AUC of AIP in T2DM patients was 0.697 (95% CI: 0.652 ~ 0.741). The optimal cut-off value was 0.182, with a sensitivity of 49.5% and a specificity of 78.5%.

## 4 Discussion

With the escalating global prevalence of diabetes, the increasing longevity, and the aging of the global population, DR has emerged as a formidable global health challenge (18). Consequently, it is imperative and promising to identify the serum biomarkers or reliable predictors for the risk of DR in T2DM populations, which might benefit from intervention in the early stage of DR and assist in decision-making in DR environment burdens.

In our study, we found that the higher TG level and the lower HDL-C level in DR groups, therefore, dyslipidemia is strongly associated with the development of DR. Similarly, a previous study reported that diabetic patients with dyslipidemia had a statistically significant increase in the incidence of DR when compared to those without dyslipidemia (19), underscoring the imperative need for integrated management strategies targeting both hyperglycemia and dyslipidemia to mitigate the risk and progression of DR. TG is the most abundant lipid in human adipose tissue, its elevated levels may lead to biotoxicity and further contribute to the development and progression of insulin resistance (3). Reversal of cholesterol transport, antioxidant, anti-inflammatory, endothelial/vasodilatory, antithrombotic and cytoprotective effects are the main biological properties of HDL-C, which plays a crucial role in the regulation of metabolic diseases (3). The AIP, calculated with the logarithm of the TG/HDL-C, was showed the dynamic levels between serum TG and HDL-C and reflected the size of lipoprotein particles, providing a more comprehensive indication of the pathogenicity and specificity of lipid abnormalities than high TG or low HDL-C levels alone. In our study, we found a significant association between elevated AIP level and increased prevalence of DR, even after adjusting for potential confounders (age, BMI, FPG, etc.). Specifically, the prevalence of DR increased from 19.6 to 56.8% with increasing AIP levels, and logistic analysis showed that when grouped by AIP quartiles, compared to the Q1 group, subjects in Q4 had a remarkably increased risk of DR (OR 4.414, 95%CI: 1.841 ~ 10.097) after adjustment for age, BMI, SBP, duration, DBIL, BUN and Cr; when AIP as a continuous variable, it was shown that AIP levels were significantly associated with DR even after adjusting for potential confounders (OR 2.804, 95%CI: 1.695 ~ 4.207). Moreover, we also assessed the diagnostic value of AIP for DR and the result showed the AUC of AIP to predict the risk of DR was 0.697 (95% CI: 0.652 ~ 0.741), suggesting that this indicator had superior clinical value in the prediction of DR in T2DM. Therefore, changes in this composite parameter should be emphasized, especially in T2DM patients with AIP higher than 0.182.

We hypothesize that the association between AIP and DR may be explained through several potential mechanisms. Firstly, in hyperlipidemia, blood viscosity increases and blood flow slows down, aggravating ischemia and hypoxia in the patient’s retinal tissues and making it easier for microthrombi to form, leading to an increased risk of DR. Although research on the correlation between AIP and DR is limited, the association between lipids and cardiovascular disease in T2DM has been extensively studied. Studies showed that AIP, as a composite index of TG and HDL levels, is closely related to cardiovascular outcomes (9). Kaze et al. found that patients with metabolic dyslipidemia had a higher risk of composite CVD outcomes and stroke compared with T2DM patients with normal TG and normal HDL-C, suggesting that attention to metabolic dyslipidemia in patients with T2DM is necessary to reduce the risk of CVD (3). What’s more, another study demonstrated that AIP could be a powerful biomarker for predicting the risk of cardiovascular events in patients with T2DM (12). Importantly, previous studies have reported a bidirectional connection between microangiopathy and CVDs in diabetic patients, the possible mechanisms of co-occurrence include accumulation of advanced glycation end products (AGEs) in the vessel wall, endothelial dysfunction, and oxidative stress, leading to alterations in the microvascular system and progression of atherosclerosis (20, 21). Thus, AIP could also be a strong predictor of microvascular complications in T2DM.

Secondly, DR and diabetic kidney disease (DKD) are both microvascular complications of DM, sharing some of the same pathological mechanisms, including endothelial damage, oxidative stress, accumulation of AGEs, and inflammatory responses induced by glycolipid metabolic disorders (22). A prospective study reported that disorders of lipid metabolism are associated with microvascular complications in patients with T2DM, and elevated AIP is associated with increased risk of DKD (23, 24). Another prospective investigation also found that AIP levels were associated with diabetic microvascular complications and suggested that AIP could be used as an independent predictor for identifying the risk of DKD in T2DM; however, the link between AIP and DR was not detected (25). Furthermore, Chen et al. found that the development of the microvascular complications of diabetes, DR and DKD, were linked with a correlation with dyslipidemia (4). The powerfully connection between DR and DKD, as well as provides additional validation to our conclusions from an alternative vantage point.

Additionally, AIP has been shown to indirectly reflect the diameter size of LDL particles and can be used to represent sdLDL size (10). SdLDL does not readily bind to plasma LDL receptors, resulting in clearance from the circulation, susceptibility to oxidation, and easy uptake by macrophages to form foam cells (26), and patients with higher levels of sdLDL are typically at higher risk for vascular complications (27). The key pathophysiology underlying the development of DR in patients with T2DM is a variety of hyperglycemia-induced changes, including thickening of the basement membrane of retinal capillaries, increased vascular permeability of the retina, tissue ischemia, and the release of a variety of vasoactive substances leading to neovascularization. Moreover, oxidative stress also plays an irreplaceable role in the pathogenesis of DR. (28) Oxidative stress induces mitochondrial damage, lipid peroxidation, retinal apoptosis and inflammation, leading to functional and structural changes in the retinal microvascular system and accelerating the process of DR. Kowluru et al. shown that impaired oxidation levels in diabetic mice with hyperlipidemia lead to increased levels of reactive oxygen species (ROS), mitochondrial dysfunction, and accelerated apoptosis of capillary cells, which adversely affects the retinal microvascular system and accelerates the development of DR, suggesting that early intervention for hyperlipidemia in diabetic patients may be an intelligent strategy to slow the progression of DR. (5) Furthermore, previous studies have reported that TCs and serum cholesterol are directly related to the incidence and severity of DR, and that long-term supplementation with the lipid-lowering therapy fenofibrate reduces the need for laser therapy in patients with proliferative DR. (29, 30) Undoubtedly, the exact mechanism between AIP and DR needs to be further elucidated.

In this study, the accuracy of utilizing AIP to predict DR in patients with T2DM was relatively remarkable, with an AUC ROC of 0.697, suggesting that this biomarker remains potentially beneficial in clinic and epidemiological studies. Besides, the critical importance of early DR diagnosis in T2DM patients, as previously discussed, further amplifies the value that AIP testing can provide. Although this study provided AIP, as a non-traditional lipid parameter, can predict of DR by the simple and easy calculation in the skyrocketing costs of health care, some limitations should be acknowledged. First, the study was a single-center cross-sectional study, and future validation in a large cohort across multiple centers is necessary to improve the generalizability and reliability of our findings. Second, our study did not demonstrate a direct causal relationship between AIP and DR. Third, although we adjusted for some of the confounders, there may still be unmeasured variables.

## 5 Conclusion

Our study elucidated the association between the risk of DR and AIP in patients with T2DM, and changes in AIP levels were correlated with the risk of DR. AIP is expected to be a new potential risk indicator to identify patients at high risk of DR at an early stage.

## Data Availability

The original contributions presented in the study are included in the article/supplementary material, further inquiries can be directed to the corresponding author.
